# Characterizing restrictions on commercial advertising and sponsorship of harmful commodities in local government policies: a nationwide study in England

**DOI:** 10.1093/pubmed/fdad155

**Published:** 2023-08-22

**Authors:** Sarah McKevitt, Martin White, Mark Petticrew, Carolyn Summerbell, Milica Vasiljevic, Emma Boyland, Steven Cummins, Anthony A Laverty, Christopher Millett, Frank de Vocht, Cornelia Junghans, Eszter P Vamos

**Affiliations:** Public Health Policy Evaluation Unit, School of Public Health, Imperial College London, London, UK; MRC Epidemiology Unit, School of Clinical Sciences, University of Cambridge, Cambridge, UK; Department of Public Health, Environments & Society, Faculty of Public Health and Policy, London School of Hygiene & Tropical Medicine, London, UK; Centre for Translational Research in Public Health, Fuse, Newcastle, UK; Department of Sport and Exercise Sciences, Durham University, Durham, UK; Centre for Translational Research in Public Health, Fuse, Newcastle, UK; Department of Psychology, Durham University, Durham, UK; Department of Psychology, University of Liverpool, Liverpool, UK; Population Health Innovation Lab, Department of Public Health, Environments & Society, London School of Hygiene & Tropical Medicine, London, UK; Public Health Policy Evaluation Unit, School of Public Health, Imperial College London, London, UK; Public Health Policy Evaluation Unit, School of Public Health, Imperial College London, London, UK; Population Health Sciences, Bristol Medical School, University of Bristol, Bristol, UK; NIHR Applied Research Collaboration Northwest London (NIHR ARC), London, UK; NIHR Applied Research Collaboration Northwest London (NIHR ARC), London, UK; Public Health Policy Evaluation Unit, School of Public Health, Imperial College London, London, UK

**Keywords:** advertisement, alcohol, commercial, commercial determinants of health, document analysis, fast foods, gambling, harmful commodity industry, HFSS, junk foods, local authority, local government, marketing, non-communicable diseases, policy, policy, population health, promotion, public health, sponsorship, sugar-sweetened beverages, tobacco, ultra-processed food, unhealthy commodity industry

## Abstract

**Background:**

Commercial advertising and sponsorship drive the consumption of harmful commodities. Local authorities (LAs) have considerable powers to reduce such exposures. This study aimed to characterize local commercial policies across all English LAs.

**Methods:**

We conducted a census of all English LAs (*n* = 333) to identify local commercial policies concerning advertising and sponsorship of tobacco, alcohol, less healthy foods and gambling, through online searches and Freedom of Information requests. We explored policy presence, commodity frequency and type, and associations with LA characteristics (region, urban/rural and deprivation).

**Results:**

Only a third (106) of LAs in England had a relevant policy (32%). These included restrictions on tobacco (91%), gambling (79%), alcohol (74%) and/or less healthy foods (24%). Policy prevalence was lowest in the East of England (22%), North East (25%) and North West (27%), higher in urban areas (36%) than rural areas (28%) and lower in the least (27%) compared with the most (38%) deprived areas. Definitions in policies varied, particularly for alcohol and less healthy foods.

**Conclusions:**

English LAs currently underutilize their levers to reduce the negative impacts of harmful commodity industry marketing, particularly concerning less healthy foods. Standardized guidance, including clarity on definitions and application, could inform local policy development.

## Background

The use and consumption of ‘harmful commodities’ (e.g. tobacco, alcohol, less healthy foods and gambling) are major drivers of adverse trends in non-communicable diseases (NCDs; e.g. obesity, type 2 diabetes and poor mental health).^1–7^ ‘Harmful Commodity Industry’ (HCI) marketing is a key mechanism of corporate influence, to increase the acceptability, desirability and consumption of products harmful to health^1^^,^^2^^,^^8–11^ with a disproportionate influence on children and lower socioeconomic groups, exacerbating health inequalities.^12–18^ HCI advertising and sponsorship undermines public health measures to reduce the burden of NCDs from behavioural risk factors.^8–11^ Addressing the exposure to HCIs is a key public health priority.^5^^,^^6^^,^^13^

Since the UK Government’s Health and Social Care Act 2012, and the devolution of public health responsibility from central to local government,^19^^,^^20^ local authorities (LAs) have substantial power and a duty to protect and promote the health of their local population.^21^ Through legislative and regulatory powers, LAs can make significant and meaningful changes to corporate activities and practices,^22^^,^^23^ including restricting advertising and sponsorship of harmful commodities in their local area. Many HCI–LA interactions, such as promotion of harmful commodities on council-owned infrastructure^24^^,^^25^ conflict with the duty to improve local population health.^21^ To respond to the current public health challenges, the government’s duty to act on the commercial determinants has gained increasing attention, and whilst many LAs have started using their levers, progress greatly varies, and it is less clear what could constitute a comprehensive strategy.

In 2019, the pioneering Transport for London (TfL) ban on high fat, salt and sugar (HFSS) food advertising inspired many LAs to implement restrictions on the marketing practices of both less healthy food and other harmful commodities across their local estate.^26^ Research has shown that such interventions are feasible^27^ and several English LAs have since implemented their own policies (e.g. Southwark Council^28^ and Bristol City Council^29^). However, whilst some LAs have adopted such policies, there is limited guidance to support LAs in how they should interact with HCIs.^30^ Little is known regarding the extent of the presence of local commercial policies across English LAs. This study aimed to characterize the presence of local policies for the advertising and sponsorship of products that are harmful to health (tobacco, alcohol, less healthy foods and gambling) across all English LAs and differences according to local area profiles (region, urban/rural classification and deprivation).

## Methods

Between July and December 2022, we conducted a hierarchical three-step process to identify and retrieve local advertising and sponsorship policies across all LAs in England (*n* = 333). We sought information concerning restrictions or considerations for four specific harmful commodities: tobacco, alcohol, less healthy foods and non-alcoholic beverages, and gambling. First, we conducted a search of all LA websites (Step 1). Second, we contacted each LA Chief Executive (CE) directly by e-mail (Step 2). Finally, we sent the remaining non-responding LAs a Freedom of Information (FOI) request (Step 3).^31^

We conducted an online document search (Step 1) that drew on methods similar to strategic searches and hand-searching grey literature.^32–35^ We developed a search strategy (Supplementary Table I) with pre-identified search terms and pilot tested it using previously identified local commercial policies.^29^ We used common search terms associated with advertising, sponsorship, guidance and policies, interchangeably and in combination, applying principles of information saturation.^36^

For each LA where the online search did not identify relevant information, we sought direct contact with the LA CE (Step 2) using an e-mail template developed with public collaborators and LA practitioners (Supplementary Material II). The e-mail introduced the research purpose and background, and requested the relevant local guidance for that specific LA. For additional clarification, we provided examples of LA policies from other areas. Immediate automated responses (CE role/contact expired or ‘failed to send’) were replaced with a new contact to address the e-mail request. We documented responses for a pragmatic period of 4 weeks.

**Fig. 1:**
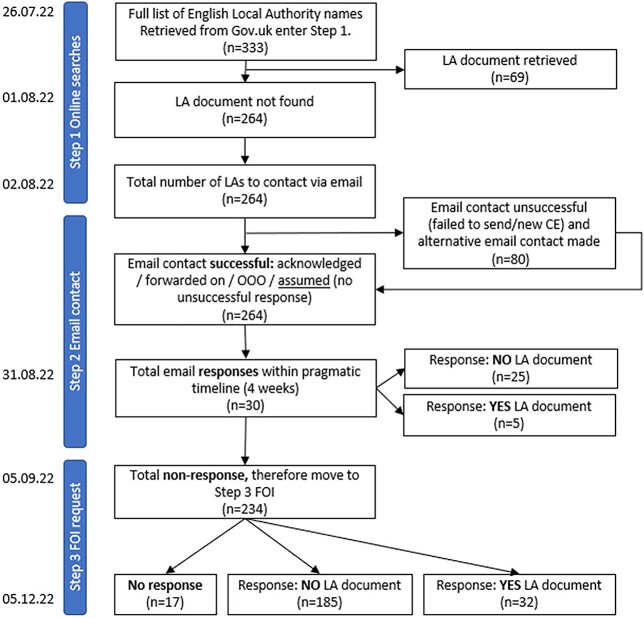
Flow diagram of data collection process and policy identification. OOO, Out Of Office.

When a conclusive response (yes/no policy) was not obtained through Steps 1 and 2, we proceeded to send FOI requests (Step 3). Public authorities are required to respond to FOI requests within 20 working days following the date of receipt.^31^ We documented responses including those explicitly articulating the absence of a local commercial policy. In the case, we did not receive an FOI response; we made a further attempt to retrieve the outstanding information via e-mails to the relevant LAs and their Directors of Public Health. After an additional grace period (December 2022), any non-responsive LAs were documented as missing data (Fig. 1).

### Data extraction

We undertook a documentary analysis to identify and describe LA policy restrictions for advertising and sponsorship of harmful commodities. Policies were included if they referred to either advertising or sponsorship as these terms were not consistently defined and often used interchangeably. We created a guiding extraction frame with both inductive and deductive interpretation to capture emergent data from the documents retrieved. Data extraction included the date and step of document identification (1/2/3), document name, year, guidance references, advertising/sponsorship spaces (e.g. billboard advertisement), number of harmful commodities considered (frequency; 0–4), harmful commodity types (tobacco, alcohol, less healthy foods and gambling), key definitions and any associated relevant contextual information. As this was an exploratory study, we included any consideration of the harmful commodities of interest but note these differ in terms of definitions and application across LAs.

To conduct subgroup analyses of policy prevalence by LA characteristics, we collected publicly available online LA data. Our subgroups of interest included English region, rural/urban classification and deprivation levels. We used the 3-fold 2011 Rural Urban Classification of the LA District boundaries, 2021^37^ to classify LA areas into three groups (predominantly urban, predominantly rural and urban with significant rural). The classification is an official statistic which uses the proportion of the population that resides in rural areas (<10 000 resident population).^37^ We used the Index of Multiple Deprivation (IMD) 2019^38^ quintiles to determine LA deprivation. The IMD is an official measure of relative deprivation in England that ranks areas according to their level of deprivation, across seven domains.^38^

### Data analysis

We used Choropleth mapping for the visual presentation of LA policy presence (yes/no), frequency of harmful commodities (0–4) and type of harmful commodities (tobacco, alcohol, less healthy foods and gambling) considered in the policies for each LA in England (Google My Maps, Map Data^©^ 2023 by Google Maps). In the case of two-tier LA structures where County Councils cover the area of multiple smaller District Councils (Supplementary Material III), we present maps of the 309 lower-tier LAs in the main analyses and show the 24 upper-tier County Councils separately as secondary analyses. We also provide additional mapping for London Boroughs. We used narrative synthesis to describe key characteristics of the LA policies, including harmful commodity definitions and policy application, and associations with LA characteristics. We estimated policy prevalence (%; proportion of LAs with a policy = number of LAs with a policy in subgroup/total number of LAs in sub-group) according to: English region, three-fold Rural Urban Classification and IMD quintiles of deprivation.

### Public involvement

Five members of the public were involved throughout the research, including planning, conduct and analysis. For example, public collaborators contributed by drafting the script for e-mail and FOI request contact and interpreting results. The public will continue to be involved in wider dissemination plans.

## Results

Out of 333 LAs, we obtained data for 314 (95%). We identified that 106 LAs (32%) had a local policy; 69 through online searches, five through e-mail responses and 32 through FOI requests (Fig. 1). There were 210 (63%) LAs without a local policy and 17 (5%) were non-responsive (Supplementary Table IV).

One-third of all English LAs had a relevant local policy in place in the period July to December 2022. A full description of all 106 policies can be found in Supplementary Table V (Parts 1 and 2). The latest date of policy publication was transparent in 57 policies and ranged from 2009 to 2022 (Supplementary Material VI). Within the 106 policies identified, only 18 (17%) considered all four harmful commodities of interest, 53 (50%) considered three, 20 (19%) considered two, 12 (11%) considered one and three (3%) reported none (but did consider, e.g. political objectives, ‘offensive’ advertisements and discrimination (e.g. religious or race)). The most common harmful commodity considered was tobacco (*n* = 96, 91%), followed by gambling (*n* = 84, 79%), alcohol (*n* = 78, 74%) and less healthy foods (*n* = 25, 24%; Table 1).

**Table 1:** Summary of commercial policy presence in English LAs

Characteristic	Frequency (*n*)	Percentage (%)
All English LAs	**333**	**100%**
Policy presence:		
Yes	106	32%
No	210	63%
Missing data (non-response)	17	5%
LAs with a policy	**106**	**100%**
Step of identification:		
Online search	69	65%
E-mail request	5	5%
FOI request	32	30%
Frequency of harmful commodities:		
0	3	3%
1	12	11%
2	20	19%
3	53	50%
4	18	17%
Type of harmful commodity:		
Tobacco	96	91%
Alcohol	78	74%
Less healthy foods	25	24%
Gambling	84	79%
Policy prevalence by region:		
East Midlands	12	31%
East of England	11	22%
London	12	36%
North East	3	25%
North West	11	27%
South East	22	31%
South West	15	46%
Yorkshire	8	36%
West Midlands	12	36%
Policy prevalence by urban/rural classification:		
Predominantly urban	64	36%
Urban with significant rural	16	26%
Predominantly rural	26	28%
Policy prevalence by deprivation quintile:		
1st least deprived	18	27%
2nd	15	23%
3rd	26	39%
4th	22	33%
5th most deprived	25	38%
Policy prevalence by LA type^a^:		
Two-tier County Councils (*n* = 24)	10	42%
Two-tier Districts (*n* = 181)	39	22%
Unitary Authorities^b^ (*n* = 59)	30	51%
Metropolitan Districts (*n* = 36)	15	42%
London Boroughs^c^ (*n* = 33)	12	36%

^a^(see Supplementary Material III)*.*

^b^Unitary Districts (*n* = 52) + Unitary Counties (*n* = 6) + Isles of Scilly (*n* = 1).

^c^London Boroughs (*n* = 32) + City of London (*n* = 1).

**Fig. 2:**
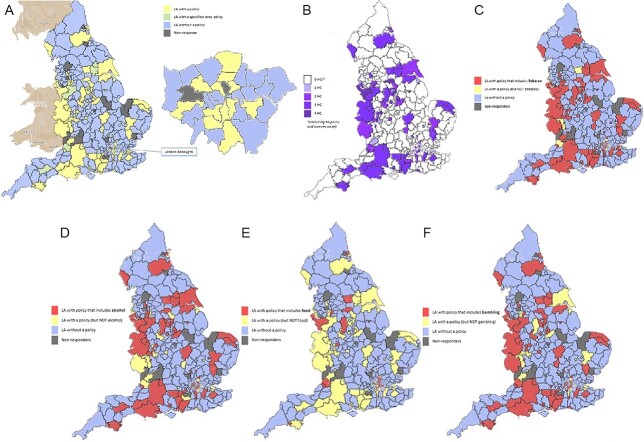
(**A**–**F**) Local commercial policy patterns according to all lower-tier English LAs. HC, harmful commodity(ies). Map source: Google My Maps using Map data 2023 Geo-Basis-DE/BKG (2009), Google.

Figure 2 presents maps displaying the patterns across all lower-tier English LAs regarding (A) policy presence, (B) harmful commodity frequency and (C–F) harmful commodity type ((C) tobacco, (D) alcohol, (E) less healthy foods and (F) gambling). Supplementary Material VII Part 1 displays the corresponding upper-tier County Council maps. Supplementary Material VII Part 2 provides an additional analysis of policy presence, whereby non-policy lower-tier LA results are replaced by upper-tier LA results for that area. Out of the 33 London borough LAs (including the City of London), 12 had a relevant local policy, 19 LAs did not have a policy and two did not respond. Supplementary Material VIII displays further London Borough maps.

### Harmful commodity definitions

Across policies, the definitions of each harmful commodity varied. Tobacco was the most clearly and consistently defined, drawing a clear line prohibiting tobacco and substitute tobacco product promotion. Some policies elaborated further with closely related details, including e-cigarettes and tobacco paraphernalia. Terminology and scope with regard to gambling was also largely consistent, with some explicit clauses for exceptions, such as the National and local society/authority lotteries. Alcohol was considered in 74% of policies but had large variation in terms of prohibited products or consumption. For example, some policies only prohibited specific alcohol scenarios: ‘encourages excessive or underage use’ and ‘binge drinking’. Furthermore, in the few policies that considered less healthy foods (24%), the UK Nutrient Profiling Model definition of HFSS food^39^ was used comprehensively in nine policies (36% of all less healthy foods considerations) but not consistently. More often, ambiguous terminology, such as ‘unhealthy eating’ or ‘fast foods’ was applied, or in specific cases: ‘fast food when promoted to minors’ or ‘not an appropriate site’ (Supplementary Table IX Parts 1 and 2). Some policies used broad clauses covering HCIs/products, or consumer behaviours: ‘socially undesirable or unhealthy acts’, ‘conflict with the wider promotion of healthy and active lifestyles’ and ‘undue publicity to inappropriate behaviour or lifestyles’. Environmental health was another common harmful commodity consideration in some policies (e.g. fossil fuels and ‘high carbon’ products).

### Policy application

Many LAs have a vast range of spaces for advertising and sponsorship in their locality. Most policies applied to all types of out-of-home advertisement and sponsorship spaces owned by the LA, from broad categories, including ‘all channels’ or ‘traditional and new media’, to specific spaces, including common advertising formats (e.g. billboards and bus shelters), to other spaces, including properties, roads and transport, green/outdoor space, community facilities and within-council communication channels (Supplementary Table X). Whilst most policies applied to most types of LA advertising/sponsorship spaces, 13 (12%) were explicitly specified area only, including bus shelters (*n* = 1), roundabouts (*n* = 5), highways (*n* = 3) and the council website (*n* = 4; Supplementary Table V Part 2). Advertisement and sponsorship constraints were often influenced by and referred to national-level regulations or guidance (Supplementary Material XI), for example, the Advertising Standards Authority UK Code of Non-broadcast Advertising, Sales Promotion and Direct Marketing (CAP code), which sets principles and guidance including alcohol and gambling behaviours.^40^

Almost two-thirds of LAs did not have any form of policy (*n* = 210, 63%). In addition, we identified some policies for private vehicle advertisements (*n* = 3), which did not cover any of the LA-owned estate, and not included in the analysis (Supplementary Table XII).

### Associations with LA characteristics

Across the nine regions of England, the two most northern regions of England and the East of England had the lowest prevalence of LA policies per LAs (<30%; Supplementary Material XIII)*.* Most policies identified were in ‘Predominantly Urban’ areas (*n* = 64, 36%), compared with ‘Predominantly Rural’ (*n* = 26, 28%) and ‘Urban with significant Rural’ areas (*n* = 16, 26%; Supplementary Material XIV). The least deprived fifth of LAs had 27% policy prevalence, compared with 38% in the most deprived. Five of the top 10 most deprived LAs had a policy covering one or more harmful commodities (Supplementary Material XV).

## Discussion

### Main findings of this study

To our knowledge, this is the first study to assess comprehensively the current presence of local advertising and sponsorship policies across all English LAs. Two-thirds of all LAs in England do not have a local policy. The 106 policies identified were heterogeneous, in application and definitions. Most often policies considered three of the four harmful commodities examined, and consistently applied tobacco restrictions. Gambling and alcohol were commonly considered but alcohol varied greatly in its definition and application. Only one quarter of policies included less healthy foods restrictions, and definitions of products were often ambiguous. Policy prevalence varied from 22 to 46% of LAs across English regions, 26 to 36% across urban rural classification and 23 to 39% according to deprivation levels. Overall, there were variations in both the presence of, and detail within, policies across the country.

### What is already known on this topic

LAs and HCIs have multifaceted relationships and interact across several LA departments (e.g. planning and transport) to generate revenue and development opportunities and enable LAs to deliver their key functions to enhance local communities.^30^ This is particularly pertinent in the current economic climate, to compensate for reduced public funding and increased financial constraints.^41–43^ However, outdoor advertising and local sponsorship are a major source of harmful commodity exposure to the public and, therefore, have the potential to shape HCI harms. The WHO recommends the best ways to prevent NCDs include interventions restricting the advertising and sponsorship of unhealthy products.^44^ Reducing exposure to unhealthy product marketing reduces their consumption,^45^ and therefore, regulatory policies are essential to create healthier local environments and improve population health.

### What this study adds

We found that two-thirds of LAs had no such policy. A previous study by Keeble *et al*.^46^ found that just half of English LAs had a takeaway food outlet planning policy and just 56 had health-specific criteria, with large variance in their content and nature. Although we identified 106 policies, these were very heterogeneous. LAs have a substantial advertising and sponsorship estate yet have a lack of consensus for harmful commodity restrictions.

Tobacco was the harmful commodity most consistently considered in local policies, likely owing to the implementation of the World Health Organization Framework Convention on Tobacco Control.^47^ Gambling was also consistently considered, which may develop further with the future Gambling Act review explicitly focused on preventing and reducing harms through marketing, advertising, promotion and sponsorship.^48^^,^^49^ However, the lack of clear cut lines and definitions, for example, concerning alcohol and less healthy foods, may introduce ambiguity for consistent targeted action. Nevertheless, with evidence for the beneficial impact of the TfL HFSS restrictions, especially in reducing inequalities,^50^^,^^51^ additional time may embed similar restrictions in practice. Likewise, more LAs are adopting the Local Authority Declaration on Healthy Weight,^52^ providing tools for LAs to promote healthy weight, which is continuing to report case studies and evaluations sharing evidence of its impact.^52^ LAs leading on the implementation of local commercial policies (e.g. Bristol City Council^53^) may serve as an exemplar for such initiatives (e.g. TfL ban and Sustain’s ‘Healthier Food Advertising Policy Toolkit’^54^), and for other LAs to implement detailed policies.

The decentralization of Public Health responsibility has provided opportunity for accelerated local actions on the commercial determinants of health (CDoH), including advertising and sponsorship policies. LAs have proven capacity to take effective action in shaping local environments to reduce the negative health impacts of HCIs.^46^^,^^55^ However, the translation of commercial policies into meaningful and feasible actions requires a consistent and clearly defined approach, which is currently lacking or suboptimal in many areas.

### Limitations of this study

We conducted a multi-step data collection process across all English LAs. We attempted data collection across all English LAs, which means our results are likely to provide an accurate reflection of current nationwide patterns of local commercial policies. To the best of our knowledge, this is the first study to provide a comprehensive assessment and characterization of such policies.

A limitation of our study is its cross-sectional design within the context of ever-changing political and health systems, and for some LAs, these policies may have evolved since we collected the data in 2022. Our findings are also contextualized in the English LA setting. Furthermore, draft, or updated documents (after December 2022), policy intentions and future emergent policies are not captured. Some LA documents that demonstrate intent but are not an actual policy (e.g. core values, health and well-being strategies) were not included in our study. In addition, we do not know the extent to which local commercial policies are implemented locally. Alternative authorities (e.g. public transport authorities) may also have relevant advertising and sponsorship policies that may considerably influence HCI marketing exposures and the policy landscape but these were not the focus of the current research.

### Research and policy implications

Although it is unclear if the presence of local policies facilitates action, they could reflect an overall approach to act on harmful commodities and to protect populations from associated harms. A consistent and clearly defined approach to regulatory policies is needed to support LAs in decision-making on minimizing the population impacts of harmful commodity marketing and promotions. This paper assessed what approaches LAs have taken, and characterized and quantified current policies. We identified that comprehensive strategies are lacking, revealing policy gaps. This research provides examples for LAs that are considering implementing policies and a baseline for future research and evaluations. Subsequent research should evaluate the impact of policies, their content and comprehensiveness according to LA profiles (e.g. deprivation) and explore policy implementation. Given the disproportionate impacts of HCIs on deprived areas, it will be important to assess the extent to which the nature and intensity of approaches are aligned with population needs. It is essential to understand potential facilitators and barriers (e.g. competing LA priorities, industry involvement/influence) towards local commercial policy adoption, including perspectives of a diverse range of stakeholders, in and outside of LAs and public health. Developing consensus and a unified approach that ensures LA policy consistency could support a wider adoption of policies by LAs for locally acceptable, meaningful and impactful population-wide action on the CDoH. In the interim, we suggest that standardized guidance, based on good practice, including clarity on definitions and application, with case study examples and training tools, be developed for England to encourage effective implementation across all LAs.

## Conclusion

Our findings suggest that two-thirds of all English LAs do not appear to have a local policy concerning advertising and sponsorship of harmful commodities in their local area. The 106 policies identified were very heterogeneous, lacking consensus regarding components, definitions and application. LAs have power to act and make significant changes to minimize negative impacts from harmful commodities but lack standardized guidance. Future research is needed to establish the most effective policy components to enable and empower LAs to act on the CDoH and improve local population health.

## Key points

- Advertising and sponsorship are key drivers of the consumption of harmful commodities and undermine public health efforts to reduce risk factors for non-communicable diseases and health inequalities.
- Local authorities (LAs) have substantial powers and levers to reduce the marketing of harmful commodities through local policies that restrict the advertising and sponsorship of harmful products in public spaces.
- Two-thirds of LAs do not currently have local policies and those that do vary in their application and definitions. Less healthy foods and beverages in particular are presently unaccounted for.
- LAs may underutilize their powers to improve health through reducing exposures to harmful commodities, and there is a lack of guidance on the optimal components, consistent definitions for harmful commodities and principles underpinning local commercial policies.

## Supplementary Material

JOPH_supplementary_fdad155

## Data Availability

The data underlying this article will be shared on reasonable request to the corresponding author.

## Notes

**Sarah McKevitt**, Dr

**Martin White**, Prof

**Mark Petticrew**, Prof

**Carolyn Summerbell**, Prof

**Milica Vasiljevic**, Dr

**Emma Boyland**, Dr

**Steven Cummins**, Prof

**Anthony A. Laverty**, Dr

**Christopher Millett**, Prof

**Frank de Vocht**, Prof

**Cornelia Junghans**, Dr

**Eszter P. Vamos**, Dr
