# Supplementary material for: Characterizing restrictions on commercial advertising and sponsorship of harmful commodities in local government policies: a nationwide study in England
Source: J Public Health (Oxf). 2023 Aug 22;45(4):878–87. doi: 10.1093/pubmed/fdad155 (PMC10687598; doi:10.1093/pubmed/fdad155)
Supplement: JOPH_supplementary_fdad155 [file joph_supplementary_fdad155.docx]

**Supplementary material**

**Article title:** Characterising restrictions on commercial advertising and sponsorship of harmful commodities in local government policies: a nationwide study in England

**Corresponding author:** Dr Sarah McKevitt, Public Health Policy Evaluation Unit, School of Public Health, Imperial College London

[s.mckevitt@imperial.ac.uk](mailto:s.mckevitt@imperial.ac.uk)

**Contents**

1. Data collection: Phase 1 Search Strategy
2. Phase 2 and Phase 3 information request template
3. English Local government structure
4. Summary of LAs with no response (missing data)
5. Description of all English LA policies

***Part 1:*** *Comprehensive policies* ***Part 2:*** *Non-comprehensive policies*

1. LA policy latest publication dates
2. ***Part 1:*** Upper-tier County Council policy maps ***Part 2:*** Policy presence across all LAs with non-policy lower-tier LA results replaced by upper-tier LA results
3. London Borough policy maps
4. Definitions

***Part 1:*** *All Harmful commodity definitions* ***Part 2:*** *Less healthy foods*

1. Type and scope of LA advertising and sponsorship spaces
2. National level guidance and regulations
3. Description of LAs that only held vehicle policies
4. LA policy prevalence by region
5. LA policy prevalence by Rural/Urban classification
6. LA policy prevalence by LA deprivation

**Supplementary Table I.** Data collection: Phase 1 Search Strategy

| Topic | Ref | Terms |
| --- | --- | --- |
| Local Authority | #1 | Full list of English LA names retrieved from GOV.UK (n=333)  AND Authority OR Council |
| Document type | #2 | Policy OR Guidance OR Principles OR Plan OR Permissions OR Strategy OR Report OR Notice OR Ban OR Ethics OR Code OR Conduct OR Permitted OR Agreement OR Regulation OR Tool OR Guide OR Framework OR Legislation OR Contract OR Pledge OR Responsibility OR Resource OR Pack |
| Key industry terms | #3 | Fast food OR HFSS OR Harmful OR Unhealthy OR Products OR Commodities OR Ultra-Processed OR Alcohol OR Food OR Sugar OR Tobacco OR Gambling OR Betting OR Smoking  OR Sex establishment OR Political OR Religious OR Loan OR Pharmaceutical |
| Commercial interactions | #4 | Commercial OR Sponsorship OR Advertisement OR Corporate OR Media OR Private OR Industry |
|  |  |  |
| *1. Google search strategy* |  | *#1 AND #2 AND (#3 OR #4)*  *Variations using search terms out of each Ref* |
| *2. LA website search tool strategy* |  | *#2 AND #4*  *#3 as a separate search* |
| *3. Search within potential documents* |  | *#2 OR #3* |

*LA: Local Authority*

**Supplementary Material II.** Phase 2 and Phase 3 information request template


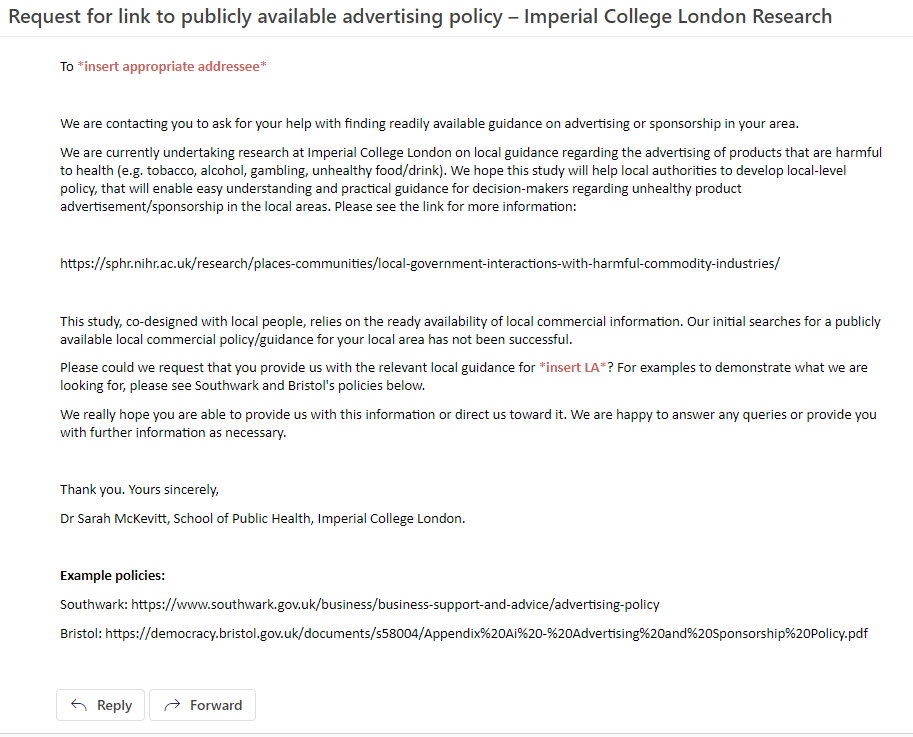


**Supplementary Material III.** English Local government structure applied in this study

**
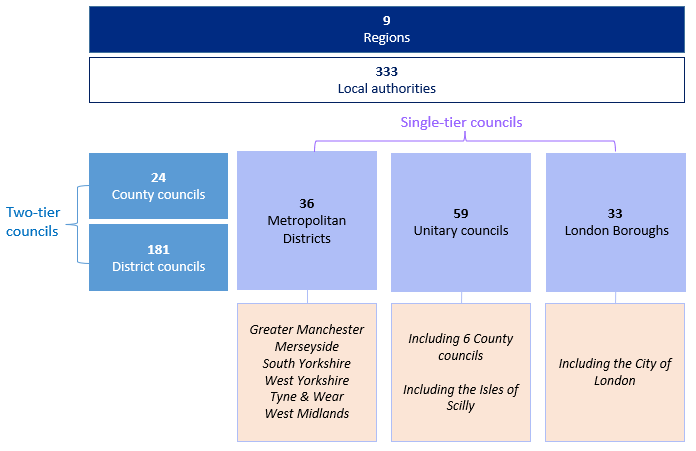
**

**Supplementary Table IV.** Summary of LAs with no response (missing data) (n=17)

|  | Local authority | Type | Region |
| --- | --- | --- | --- |
| 1 | Bolton | Metropolitan District | North West |
| 2 | Colchester | Two-Tier District | East of England |
| 3 | Cotswold | Two-Tier District | South West |
| 4 | Dacorum | Two-Tier District | East of England |
| 5 | Darlington | Unitary District | North East |
| 6 | Ealing | London Borough | London |
| 7 | Forest of Dean | Two-Tier District | South West |
| 8 | Guildford | Two-Tier District | South East |
| 9 | Halton | Unitary District | North West |
| 10 | Hastings | Two-Tier District | South East |
| 11 | Islington | London Borough | London |
| 12 | King's Lynn And West Norfolk | Two-Tier District | East of England |
| 13 | Leicester | Unitary District | East Midlands |
| 14 | Newcastle upon Tyne | Metropolitan District | North East |
| 15 | Oxford | Two-Tier District | South East |
| 16 | Ribble Valley | Two-Tier District | North West |
| 17 | South Kesteven | Two-Tier District | East Midlands |

**Supplementary Table V.** Description of all English LA policies (n=106)

| ***Part 1:*** *Comprehensive policies (****n=93)*** | | | | | | | | | |
| --- | --- | --- | --- | --- | --- | --- | --- | --- | --- |
| **Local authority** | **Type** | **Phase** | **Name of document** | **Year** | **No#** | **Tob** | **Gamb** | **Alc** | **Food** |
| Barnsley | Metropolitan District | 1 | 1. Advertising and Sponsorship Policy  2. HFSS Policy Guidance Note | 2022 | 4 | Yes | Yes | Yes | Yes |
| Basingstoke and Deane | Two-Tier District | 1 | Advertising and Sponsorship Policy | ? | 3 | Yes | Yes |  |  |
| Blackburn with Darwen | Unitary District | 1 | Advertising Policy | 2017 | 3 | Yes | Yes | Yes |  |
| Blackpool | Unitary District | 1 | Advertising Policy | 2021 | 1 | Yes |  |  |  |
| Bolsover | Two-Tier District | 1 | Advertising and Sponsorship Policy DRAFT | 2013 | 3 | Yes | Yes | Yes |  |
| Bournemouth, Christchurch and Poole | Unitary District | 1 | Advertising governance policy: Communications and Marketing | 2022 | 3 | Yes | Yes | Yes |  |
| Braintree | Two-Tier District | 1 | Advertising & Sponsorship Opportunities | 2018 | 3 | Yes | Yes |  |  |
| Brent | London Borough | 3 | Sponsorship and Advertising Code | ? | 3 | Yes | Yes | Yes |  |
| Bristol | Unitary District | 1 | 1. Advertising and Sponsorship Policy 2. High Fat, Salt or Sugar Policy Guidance Note | 2018 | 4 | Yes | Yes | Yes | Yes |
| Broadland | Two-Tier District | 1 | Financial Performance and Direction: Advertising and Sponsorship Policy | 2014 | 3 | Yes | Yes | Yes |  |
| Bromsgrove | Two-Tier District | 1 | Sponsorship and Advertising Policy | 2016 | 1 | Yes |  |  |  |
| Broxbourne | Two-Tier District | 1 | Advertising policy Code of Practice | ? | 3 | Yes | Yes | Yes |  |
| Cambridge | Two-Tier District | 1 | Sponsorship Policy and Procedures | 2009 | 1 | Yes |  |  |  |
| Cambridgeshire | Two-Tier County | 2 | Advertising and Sponsorship Policy | 2018 | 3 | Yes | Yes | Yes |  |
| Camden | London Borough | 3 | Advertising Policy | ? | 1 |  |  |  | Yes |
| Cheltenham | Two-Tier District | 1 | Corporate Advertising & Sponsorship | 2012 | 1 | Yes |  |  |  |
| Cheshire West and Chester | Unitary District | 2 | Advertising & sponsorship Policy | 2021 | 4 | Yes | Yes | Yes | Yes |
| Chichester | Two-Tier District | 3 | Advertising and sponsorship opportunities | 2022? | 3 | Yes | Yes | Yes |  |
| Copeland | Two-Tier District | 1 | Advertising and Sponsorship Policy | 2017 | 3 | Yes | Yes | Yes |  |
| County Durham | Unitary County | 3 | Sponsorship and advertising Procedure and Guidance | 2012 | 3 | Yes | Yes | Yes |  |
| Coventry | Metropolitan District | 3 | Advertising and Sponsorship Policy | ? | 2 | Yes | Yes |  |  |
| Crawley | Two-Tier District | 1 | Advertising & Sponsorship Policy | ? | 3 | Yes | Yes | Yes |  |
| Croydon | London Borough | 1 | Advertisements displayed in the Town Centre | 2013 | 0 |  |  |  |  |
| Derby | Unitary District | 1 | Policy for generating income through advertising and sponsorship | 2021 | 3 | Yes | Yes |  |  |
| Derbyshire | Two-Tier County | 1 | Income generation through advertising - Advertising and Sponsorship Policy | 2019 | 4 | Yes | Yes | Yes | Yes |
| Derbyshire Dales | Two-Tier District | 1 | Sponsorship guidelines | ? | 3 | Yes |  | Yes | Yes |
| Devon | Two-Tier County | 1 | Advertising and sponsorship policy | ? | 4 | Yes | Yes | Yes | Yes |
| Dorset Council | Unitary District | 1 | Sponsorship and Advertising Guidelines | 2020 | 3 | Yes |  |  |  |
| Dudley | Metropolitan District | 3 | Commercial advertising and sponsorship guidance | ? | 3 | Yes | Yes | Yes |  |
| East Hampshire | Two-Tier District | 1 | Advertising Policy | ? | 3 | Yes | Yes | Yes |  |
| East Riding of Yorkshire | Unitary District | 2 | Advertising and Sponsorship protocol | ? | 2 | Yes |  |  |  |
| East Staffordshire | Two-Tier District | 1 | Sponsorship opportunities: Media Pack | ? | 2 | Yes | Yes |  |  |
| Eastleigh | Two-Tier District | 1 | Terms and conditions: Advertising and Sponsorship Policy | ? | 3 | Yes | Yes | Yes |  |
| Enfield | London Borough | 3 | Advertising Policy | ? | 1 |  |  |  | Yes |
| Epsom and Ewell | Two-Tier District | 1 | Advertising Policy | ? | 3 | Yes | Yes | Yes |  |
| Gedling | Two-Tier District | 1 | Advertising and sponsorship policy | ? | 3 | Yes | Yes | Yes |  |
| Greenwich | London Borough | 3 | RBG Ad Policy: Approval Guidance Food and Non-Alcoholic Drink advertising, including breastmilk substitutes | 2021 | 1 |  |  |  | Yes |
| Hackney | London Borough | 3 | Hackney Council’s Corporate Advertising and Sponsorship Policy 2022 | 2022 | 4 | Yes | Yes |  | Yes |
| Hampshire | Two-Tier County | 1 | Advertising policy | 2019 | 3 | Yes |  | Yes |  |
| Haringey | London Borough | 1 | Corporate advertising and sponsorship Policy | 2019 | 4 | Yes | Yes | Yes | Yes |
| Hart | Two-Tier District | 3 | Draft Advertising and sponsorship policy | 2017-2022 | 3 | Yes | Yes | Yes |  |
| Havant | Two-Tier District | 1 | Advertising policy | ? | 3 | Yes | Yes | Yes |  |
| Herefordshire | Unitary District | 1 | Communications protocols and principles | ? | 2 | Yes | Yes |  |  |
| Hounslow | London Borough | 1 | Advertising code of conduct | ? | 3 | Yes | Yes | Yes |  |
| Lambeth | London Borough | 1 | Advertising and Sponsorship Policy | 2020 | 4 | Yes | Yes |  |  |
| Leeds | Metropolitan District | 1 | Advertising content guidance | 2010 | 3 |  |  |  |  |
| Lincoln | Two-Tier District | 1 | Advertising policy | ? | 4 | Yes | Yes | Yes |  |
| Lincolnshire | Two-Tier County | 1 | Advertising and Sponsorship Policy | 2020 | 2 | Yes | Yes |  |  |
| Liverpool | Metropolitan District | 1 | Corporate sponsorship (incoming) policy | 2015 | 3 | Yes | Yes |  |  |
| Luton | Unitary District | 3 | Advertising and Sponsorship Policy | 2021 | 4 | Yes | Yes |  |  |
| Maidstone | Two-Tier District | 1 | Culture and Leisure Sponsorship and Advertising Policy | ? | 3 | Yes | Yes |  |  |
| Malvern Hills | Two-Tier District | 1 | Sponsorship & Advertising policy | 2013 | 0 |  |  |  |  |
| Manchester | Metropolitan District | 3 | Contractual Clauses | ? | 3 | Yes | Yes |  | Yes |
| Medway | Unitary District | 1 | Advertising and Sponsorship Policy | ? | 1 | Yes |  |  |  |
| Merton | London Borough | 1 | 1. Advertising and Sponsorship Protocol 2. Advertising HFSS Food and Non-Alcoholic Drink Targeted At Children | 2020 | 4 | Yes | Yes | Yes | Yes |
| Norfolk | Two-Tier County | 1 | Advertising Framework | 2014 | 4 | Yes | Yes | Yes | Yes |
| North East Derbyshire | Two-Tier District | 1 | Advertising and Sponsorship Policy | 2017 | 3 | Yes | Yes | Yes |  |
| North Lincolnshire | Unitary District | 1 | Commercial advertising & sponsorship policy | 2016 | 3 | Yes | Yes | Yes |  |
| North Norfolk | Two-Tier District | 1 | Sponsorship and Advertising Policy | 2020 | 3 | Yes | Yes |  |  |
| North Northamptonshire | Unitary District | 1 | Policy for generating income through advertising and sponsorship | ? | 3 | Yes | Yes |  |  |
| North Tyneside | Metropolitan District | 2 | Advertising and publication protocol | 2022 | 4 | Yes | Yes | Yes | Yes |
| Nottingham | Unitary District | 3 | Advertising & Sponsorship Policy | 2016 | 3 | Yes | Yes | Yes |  |
| Nottinghamshire | Two-Tier County | 1 | Advertising and Sponsorship Policy | ? | 2 | Yes | Yes |  |  |
| Plymouth | Unitary District | 3 | Advertising Policy | 2022? | 4 | Yes | Yes | Yes |  |
| Portsmouth | Unitary District | 2 | Advertising and sponsorship policy | 2021 | 3 | Yes |  | Yes |  |
| Reading | Unitary District | 1 | Advertising and Sponsorship Policy | ? | 1 |  | Yes |  |  |
| Rotherham | Metropolitan District | 1 | Advertising and Sponsorship Policy | ? | 2 | Yes | Yes |  |  |
| Rushcliffe | Two-Tier District | 1 | Sponsorship and advertising opportunities | 2011 | 2 | Yes |  | Yes |  |
| Rushmoor | Two-Tier District | 1 | Advertising policy | 2016 | 3 | Yes | Yes | Yes |  |
| Salford | Metropolitan District | 3 | Advertising Content guidance | ? | 2 | Yes |  | Yes |  |
| Sefton | Metropolitan District | 1 | Sponsorship Policy | 2022 | 1 |  | Yes |  |  |
| Sevenoaks | Two-Tier District | 3 | Paid for advertising policy | ? | 3 | Yes | Yes | Yes |  |
| Shropshire | Unitary County | 1 | 1. Asset sponsorship scheme 2. Advertising policy (appendix) | ? | 3 | Yes | Yes | Yes |  |
| South Hams | Two-Tier District | 1 | Sponsorship Framework | ? | 3 | Yes | Yes | Yes |  |
| South Somerset | Two-Tier District | 1 | Advertising Policy | 2015 | 3 | Yes | Yes | Yes |  |
| South Tyneside | Metropolitan District | 3 | Advertising and Sponsorship Policy | ? | 2 | Yes | Yes |  |  |
| Southampton | Unitary District | 1 | Advertising guidance | ? | 3 | Yes | Yes | Yes |  |
| Southwark | London Borough | 1 | Advertising policy | 2019 | 4 | Yes | Yes | Yes | Yes |
| Stevenage | Two-Tier District | 1 | Corporate Advertising and Sponsorship Framework | 2021 | 3 | Yes | Yes |  |  |
| Stroud | Two-Tier District | 1 | Advertising & Sponsorship Policy | 2015 | 1 | Yes |  |  |  |
| Surrey | Two-Tier County | 1 | Advertising and Sponsorship Policy | 2019 | 0 |  |  |  |  |
| Surrey Heath | Two-Tier District | 1 | Advertising policy | ? | 3 | Yes | Yes | Yes |  |
| Swindon | Unitary District | 1 | Media Pack | 2021 | 3 | Yes | Yes | Yes |  |
| Telford and Wrekin | Unitary District | 3 | Policy for generating income through advertising and sponsorship | ? | 3 | Yes | Yes |  |  |
| Trafford | Metropolitan District | 3 | Advertising Policy | 2013 | 2 | Yes |  | Yes |  |
| Walsall | Metropolitan District | 1 | Advertising and sponsorship policy | 2018 | 4 | Yes | Yes | Yes | Yes |
| Wandsworth | London Borough | 1 | Advertising policy | 2019 | 3 | Yes | Yes | Yes |  |
| Waverley | Two-Tier District | 1 | Sponsorship policy | 2019 | 2 | Yes | Yes |  |  |
| West Suffolk | Two-Tier District | 1 | Sponsorship and advertising policy | 2015 | 2 | Yes |  |  |  |
| Wiltshire | Unitary County | 1 | Third Party Advertising Policy | ? | 3 | Yes | Yes |  |  |
| Wirral | Metropolitan District | 1 | Advertising policy | ? | 4 | Yes | Yes |  |  |
| Wolverhampton | Metropolitan District | 3 | Sponsorship and Advertising Policy | 2022 | 2 | Yes | Yes |  |  |
| Wyre Forest | Two-Tier District | 1 | Advertising and sponsorship | ? | 2 | Yes |  | Yes |  |

| ***Part 2:*** *Policies included but not comprehensive* (e.g. only cover vehicles, roundabouts, non-local information but it is a legitimate policy)* ***(n=13)*** | | | | | | | | | | |
| --- | --- | --- | --- | --- | --- | --- | --- | --- | --- | --- |
| **Local authority** | **Type** | **Phase** | **Name of document** | **Year** | ***Types/spaces covered** | **No#** | **Tob** | **Gamb** | **Alc** | **Food** |
| Brighton and Hove | Unitary District | 1 | Advertising restrictions (information cited directly from Clear Channel only) | ? | Advertising in respect of bus shelters | 2 | Yes | Yes |  |  |
| Cheshire East | Unitary District | 3 | Page 25: interim roundabout advertising policy | 2021 | Roundabouts | 3 | Yes | Yes | Yes |  |
| Essex | Two-Tier County | 1 | Advertising and Sponsorship Policy (Highways and Transport) | 2020 | Lists beyond highway: Events, activities, campaigns, facilities, assets or initiatives | 2 | Yes |  | Yes |  |
| Mid Devon | Two-Tier District | 1 | Sponsorship of and advertising on Highway Assets | 2018 | Highway | 3 | Yes | Yes | Yes |  |
| Milton Keynes | Unitary District | 3 | Roundabout sponsorship | 2022 | Roundabouts | 2 | Yes | Yes |  |  |
| North East Lincolnshire | Unitary District | 3 | Sponsorship restrictions roundabouts | ? | Roundabouts | 1 | Yes |  |  |  |
| Reigate and Banstead | Two-Tier District | 1&2 | 1. Roundabout sponsorship terms and conditions | ? | Roundabouts | 2 | Yes |  | Yes |  |
| Selby | Two-Tier District | 3 | Roundabout advertising policy | ? | Roundabouts | 3 | Yes | Yes | Yes |  |
| Somerset | Two-Tier County | 3 | Highways Advertising Policy Advertising Policy Exclusions and Permissions | 2022 | Lists beyond highway: publications, promotional materials, events, property, assets, website, roundabouts, fleet vehicles | 4 | Yes | Yes |  |  |
| South Gloucestershire | Unitary District | 1&3 | Advertising on the council website | ? | Website | 2 |  | Yes |  |  |
| Torbay | Unitary District | 1 | Website advertising policy | ? | Website only | 3 | Yes | Yes | Yes |  |
| Worcester | Two-Tier District | 1&3 | 1. Website advertising policy 3. Advertising/sponsorship requirements | ? | Website only | 3 | Yes | Yes | Yes |  |
| York | Unitary District | 3 | Advertising policy | ? | Website | 3 | Yes | Yes | Yes |  |

***Notes:*** *Green: Yes this harmful commodity is considered in LA policy; Amber shading: Harmful commodity included but with a more ambiguous harmful commodity definition; Red: No this harmful commodity is not considered in LA policy. Phase: (1) online searches, (2) email, (3) Freedom Of Information. ?: unknown; No#: Number(frequency) of harmful commodities considered in LA policy; Tob: Tobacco; Gamb: Gambling; Alc: Alcohol; Food: less healthy foods; HFSS: High in Fat Salt and Sugar.*

**Supplementary Table VI.** LA policy latest publication dates (when available) (n=57)

| **Year** | **2009** | **2010** | **2011** | **2012** | **2013** | **2014** | **2015** | **2016** | **2017** | **2018** | **2019** | **2020** | **2021** | **2022** |
| --- | --- | --- | --- | --- | --- | --- | --- | --- | --- | --- | --- | --- | --- | --- |
| **Freq** | 1 | 1 | 1 | 2 | 4 | 2 | 4 | 4 | 3 | 5 | 7 | 6 | 9 | 8 |

**Supplementary material VII. Part 1.** Upper-tier County Council (n=24) policy maps (A-F)

1. Map displaying the presence of local advertising/sponsorship policies in all upper-tier County Councils (n=24)
2. Map displaying the frequency of harmful commodity (HC) (0-4) considerations local advertising/sponsorship policies in all upper-tier County Councils (n=24)
3. Map displaying the presence of tobacco considerations in local policies in all upper-tier County Councils
4. Map displaying the presence of alcohol considerations in local policies in all upper-tier County Councils
5. Map displaying the presence of less healthy foods (‘food’) considerations in local policies in all upper-tier County Councils
6. Map displaying the presence of gambling considerations in local policies in all upper-tier County Councils


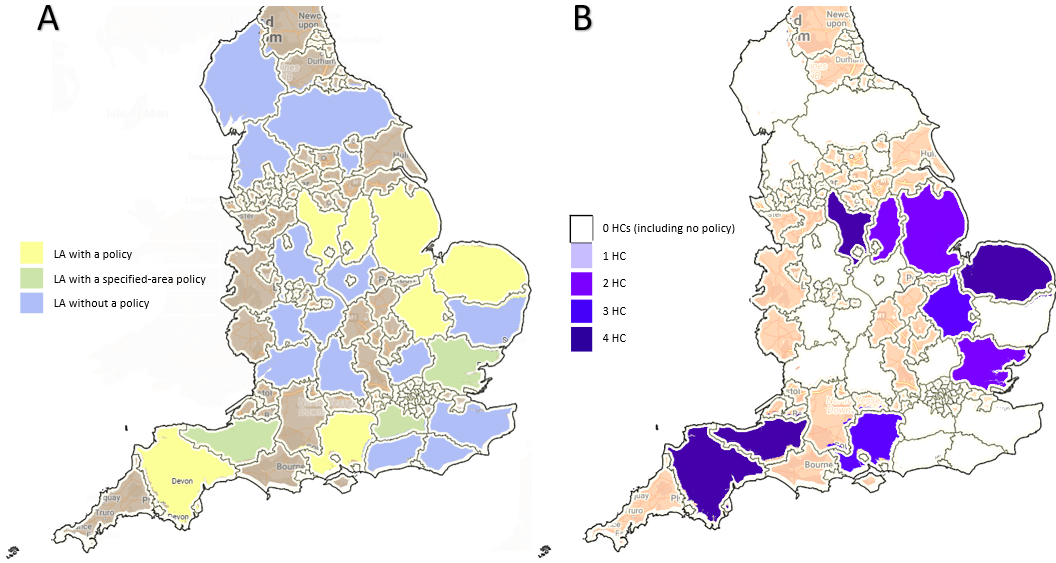


**
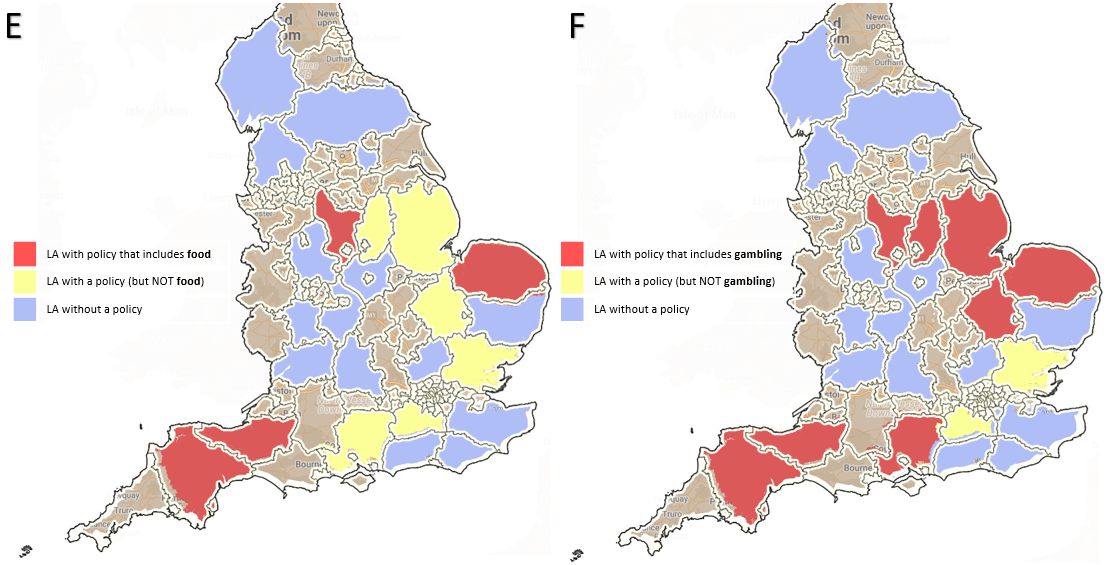

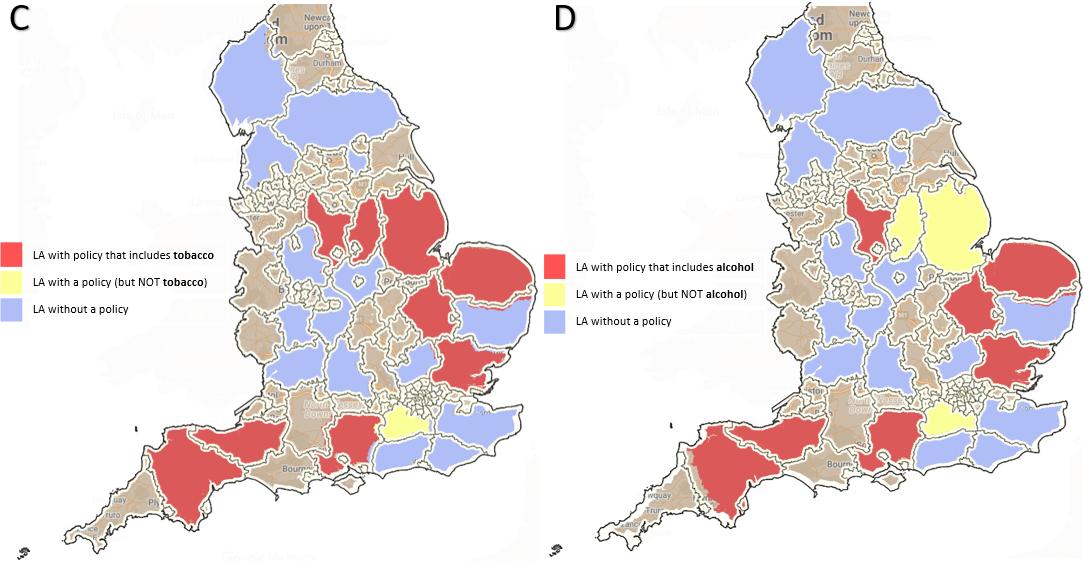
**

***Map source:*** *Google My Maps using* *Map data 2023 Geo-Basis-DE/BKG (2009), Google.* ***County Council boundaries sourced from:*** *Office for National Statistics Licensed under the Open Government Licence v.3.0 Contains OS data Crown copyright and database right 2022.*

**Supplementary material VII. Part 2.** Policy presence across all LAs with non-policy lower-tier LA results replaced by upper-tier LA results

The DOH^1^ summarise the new public health role of LAs since the Health and Social Care Act 2012 as giving ‘responsibility for health improvement to upper tier and unitary authorities’. Upper tier authorities will be supported in this by the existing expertise within district councils. Upper tier and unitary LAs therefore have public health responsibilities. They are required to appoint an individual responsible for public health functions – the Director of Public Health. The Department for Communities and Local Government^2^ states that throughout England, local planning authorities are responsible for the advertisement control system, normally the district or county council.

1. *Public Health in Local Government. 2012. The new public health role of local authorities. Department of health. Produced by the Department of Health.* [*https://assets.publishing.service.gov.uk/government/uploads/system/uploads/attachment_data/file/213009/Public-health-role-of-local-authorities-factsheet.pdf*](https://assets.publishing.service.gov.uk/government/uploads/system/uploads/attachment_data/file/213009/Public-health-role-of-local-authorities-factsheet.pdf)
2. *Communities and Local Government. 2007. Outdoor advertisements and signs: a guide for advertisers. Department for Communities and Local Government.* [*https://assets.publishing.service.gov.uk/government/uploads/system/uploads/attachment_data/file/11499/326679.pdf*](https://assets.publishing.service.gov.uk/government/uploads/system/uploads/attachment_data/file/11499/326679.pdf)

**Table 1.** Lower-tier district councils that had no/missing policy but are potentially covered by the seven upper-tier county councils with a **full policy** (n=35).

| **Cambridgeshire** | **Devon** | **Derbyshire** | **Hampshire** | **Lincolnshire** | **Norfolk** | **Nottinghamshire** |
| --- | --- | --- | --- | --- | --- | --- |
| East Cambridgeshire  Fenland  Huntingdonshire  South Cambridgeshire | East Devon  North Devon  Teignbridge  Torridge  West Devon | Amber Valley  Chesterfield  Erewash  High Peak  South Derbyshire | Fareham  Gosport  New Forest  Test Valley  Winchester | Boston  East Lindsey  North Kesteven  South Holland  South Kesteven  West Lindsey | Breckland  Great Yarmouth  Kings Lynn and West Norfolk  Norwich  South Norfolk | Ashfield  Bassetlaw  Broxtowe  Mansfield  Newark and Sherwood |

*Top: 7 upper-tier LAs with a full policy. Bottom: 35 lower-tier LAs that had no/missing policy in these upper-tier areas.*

**Table 2.** Lower-tier district councils that had no/missing policy but are potentially covered by the three upper-tier county councils with a **specified-area only policy** (n=21).

| **Essex** | **Surrey** | **Somerset** |
| --- | --- | --- |
| Basildon  Brentwood  Castle Point  Chelmsford  Colchester  Epping Forest  Harlow  Maldon  Rochford  Tendring  Uttlesford | Elmbridge  Guildford  Mole Valley  Runnymede  Spelthorne  Tandridge  Woking | Mendip  Sedgemoor  Somerset West and Taunton |

*Top: 3 upper-tier LA with a specified-area only policy. Bottom: 21 lower-tier LAs that had no/missing policy in these upper-tier areas.*

*
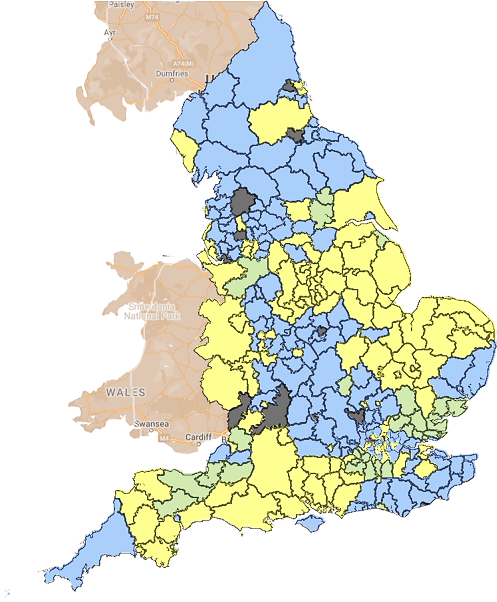
*

**Figure.** Map displaying the presence of local advertising/sponsorship policies in all lower-tier English local authorities (n=309) with non-policy lower-tier LAs overruled by upper-tier LA results.

***Notes:*** *If a lower-tier LA had no/missing (grey) or a specified-area only (green) policy, but the upper-tier LA for the same area did have a policy, the lower-tier are replaced with ‘yes policy’ (yellow). If a lower-tier LA had no/missing policy (grey), but the upper-tier LA for the same area had a specified-area only policy (green), the lower-tier are replaced with ‘specified-area policy’ (green). If an upper-tier LA also had no/missing policy, we* ***did not*** *change the lower-tier policy results.*

***Map source:*** *Google My Maps using* *Map data 2023 Geo-Basis-DE/BKG (2009), Google.*


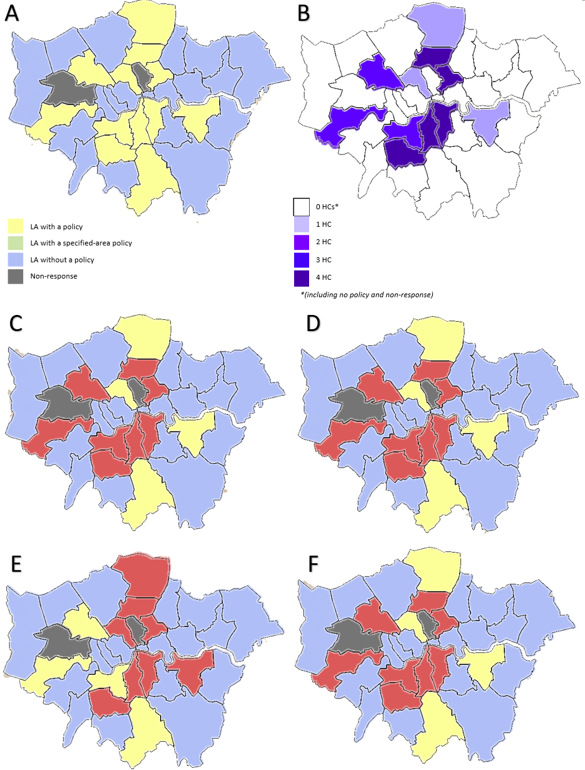

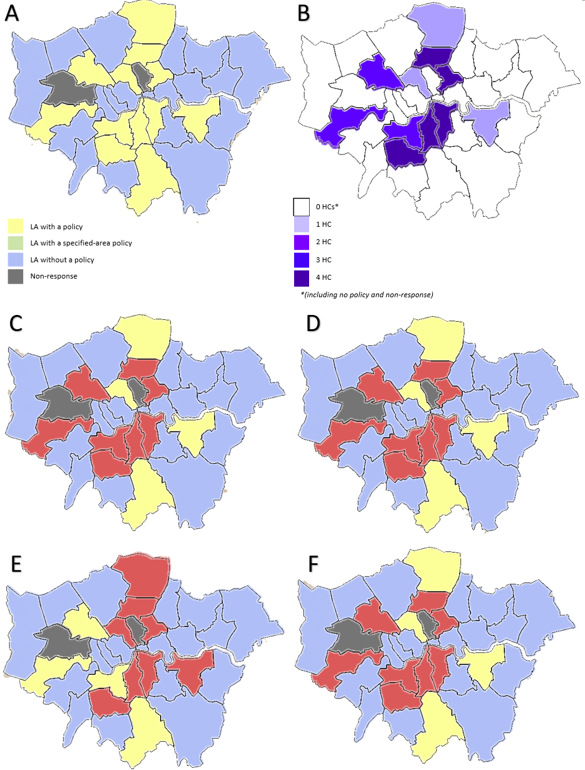
**Supplementary material VIII.** London Borough (n=33) policy maps (A-F)


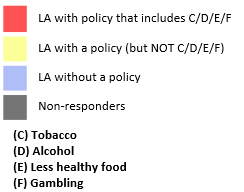

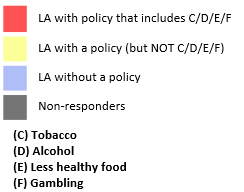


***Map source:*** *Google My Maps using* *Map data 2023 Geo-Basis-DE/BKG (2009), Google.*

**Supplementary Table IX. Part 1.** LA policy harmful commodity definitions

| **Harmful commodity** | **Definition** |
| --- | --- |
| ***Best practice (most consistently applied definitions)*** | |
| Tobacco | ‘Promotion of tobacco products or substitute tobacco products’ |
| Gambling | ‘Promotes gambling or betting, except licenced or registered lotteries benefiting local good causes e.g. society or local authority lotteries’ |
| Alcohol | ‘Promotion of alcoholic drinks. This includes advertisements where there is a range of drink featured, some of which are alcoholic’ |
| Less healthy foods | ‘Promotion of foods and drinks that are high in fat, salt and/or sugar (HFSS) as defined by the nutrient profiling model. This includes advertisements where there is a range of food/drink featured, some of which is HFSS.’ |
| ***Common alternative definitions and scope (alcohol and less healthy foods only)*** | |
| Alcohol | 'Promotes the misuse of alcohol or promotes the use of alcohol to children' |
|  | ‘Promotes the irresponsible consumption of alcohol’ |
|  | ‘Irresponsible use of alcohol’ |
|  | ‘Manufacturers and distributors of alcohol…Alcohol when promoted for irresponsible consumption' |
|  | ‘Alcohol: when the sponsorship is related to children, youth or sport’ |
|  | ‘Producers of alcoholic products are not excluded, particularly local based industries, advertisements of the alcoholic products themselves will not be permitted.’ |
|  | ‘Alcohol - where it encourages excessive or underage use’ |
|  | ‘Proximity of advertisements, not unduly promote consumption of alcohol, nor 'binge drinking' or drinks likely to appeal to young people’ |
| Less healthy foods | 'Fast food when promoted to minors' |
|  | ‘Advertising of foods and drink will be restricted to products and services that either support or are related to healthy behaviours. ‘Healthy behaviours will be determined using current health and wellbeing guidance (www.nhs.uk)’ |
|  | 'Unhealthy eating' (no further detail) |
|  | ‘Fast foods’ (no further detail) |
|  | ‘Not an appropriate site advertising of “junk foods” e.g. adjacent to schools and play areas.’ |
|  | ‘The advertisement is situation inappropriate, for example adverts for fast food or alcohol in leisure centres’ |

**Supplementary Table IX. Part 2.** Less healthy foods definitions

| **Local authority** | **Less healthy foods definition/scope** |
| --- | --- |
| Barnsley | HFSS NPM |
| Bristol | HFSS NPM |
| Camden | States HFSS (no elaboration) |
| Cheshire West and Chester | Junk food or high in HFSS (no elaboration) |
| Derbyshire | HFSS NPM |
| Derbyshire Dales | Unhealthy food |
| Devon | Junk food |
| Enfield | HFSS NPM |
| Greenwich | HFSS NPM |
| Hackney | HFSS NPM |
| Haringey | HFSS NPM |
| Lambeth | Fast food – promoted to minors |
| Leeds | Junk food – inappropriate location |
| Lincoln | Food restricted to supporting healthy behaviours |
| Luton | Adopt the approach agreed in its new HFSS policy (not identified) |
| Manchester | Less healthy (refers to NPM) |
| Merton | HFSS NPM |
| Norfolk | Food restricted to supporting healthy behaviours |
| North Tyneside | States HFSS (no elaboration) promoted to minors |
| Plymouth | Junk food that do not support healthy behaviours |
| Somerset | Unhealthy eating |
| South Gloucestershire | Fast food |
| Southwark | HFSS NPM |
| Walsall | Fast food considered unhealthy |
| Wirral | Fast food – inappropriate location |

| **Summary of less healthy foods definition frequency** | | | | |
| --- | --- | --- | --- | --- |
| HFSS NPM comprehensive | HFSS or NPM incomprehensive | Junk / fast / unhealthy food | Location specific | Minors only |
| 9 (36%) | 4 (16%) | 8 (32%) | 2 (8%) | 2 (8%) |

*HFSS: High in fat, salt and sugar; NPM: the nutrient profiling model*

**Supplementary Table X.** Policy application (advertising/sponsorship spaces)

| Type | Scope |
| --- | --- |
| Broad clauses | Goods, services, ideas, causes, opportunities, gifts; All forms, print and electronic; All paid-for advertising and sponsorship on all channels (external and internal); Companies owned that occupy or provide services; Services the council is responsible; Traditional and new media |
| Property | Council leased premises; LA is landlord – billboards, hoardings, public transport  Outdoor; Property – owned, controlled, operated, or managed by LA whether real (building) or moveable; Wholly-owned buildings and outdoor sites |
| Town and city | Advertising frames; Banners; Billboards; Digital screens; Dog bins; Flyers; Hoardings; Notice boards; Posters; Signs; Street furniture; Toilets; Waste bins |
| Roads/transport | Barriers; Boundary signs; Bridges; Bus shelters; Car parks; Central reservations; Park & ride buses; Recycling/waste service vehicles; Roundabouts; Streetlight/lamppost banners; Traffic islands; Transport; Vehicles |
| Green/outdoor space | Embankment/verges; Flower beds; Fountains; Gardens; Grass verge; Hanging baskets; Land; Landscapes; Open/green spaces; Parks; Planter signs; Public spaces |
| Printed | Brochure; Car park tickets; Council tax billing information; Direct mail; Magazines; Museum/arts/events/exhibition programmes; Newspapers; Tickets |
| Digital | Blogs; Radio; Social media; Television; Text messages |
| Community | Airport; Awards, prizes, gifts; Christmas lights; Entertainment; Homes; Leisure Centres; Libraries; Performances; Schools; Shows; Sporting; Tourism; Waterpark |
| Within council | Communication channels; Corporate and service publications; Council event/campaign/initiative; E-mails; E-newsletters; Intranet; Objects at meeting/seminar/fair/event/performance; Objectives at meetings; Presentations; Project; Purchase; Screensavers; Seminar; Sponsorship of individual/event/organisation/project; Uniforms; Websites; Workshops |

**Supplementary Material XI.** National-level regulations or guidance

- Advertising Standards Authority
- Advertising Standards board of Finance (ASBOF)
- British Code of Advertising – Sales, Promotion and Direct Marketing
- Consumer Protection from Unfair Trading Regulations 2008
- Equality Act 2010
- Financial Services Authority
- Government Tackling Obesity Strategy
- Human Rights Act 1998
- Local Government Declaration on Sugar Reduction and Healthier Foods
- Local Government Publicity
- National parks UK (NPUK) Sponsorship Policy
- Office for Health Improvement and Disparities (OHID) recommendations for sugar and calorie reduction
- Periodical Publishers Association (PPA)
- Public Health England - Nutrient Profiling Model (NPM)
- Public Regulations 2015
- Transport for London Advertising Policy – effective 25 February 2019
- TFL Advertisement Policy – Approval Guidance Food and Non-Alcoholic Drink Advertising 2019
- The Code of recommended practice on Local Authority publicity (LAP)
- The Eastern Shires purchasing Organisation (ESPO) Framework
- The National Planning Policy Framework (NPPF)
- Town and Country Planning Act (Control of Advertisements) (England) regulations 2007
- UK Code of Non-Broadcast Advertising, Sales Promotion and Direct Marketing (CAP code) and Broadcast Media (BCAP code)
- World Health Organisation (WHO)

**Supplementary Table XII.** Description of LAs that only held vehicle policies (Hackney Carriage, private hire) and **not included** in analysis (n=3)

| **Local authority** | **Type** | **Phase** | **Name of document** | **Year** | ***Types/spaces covered** | **No#** | **Tob** | **Gamb** | **Alc** | **Food** |
| --- | --- | --- | --- | --- | --- | --- | --- | --- | --- | --- |
| Breckland | Two-Tier District | 1 | Advertising on Vehicles | 2022 | Advertising on vehicles (taxi/licensed vehicles) | 3 | Yes | Yes | Yes |  |
| Chesterfield | Two-Tier District | 3 | Hackney Carriage and private hire vehicles licensing policy | ? | Vehicles | 2 | Yes |  | Yes |  |
| Tendring | Two-Tier District | 3 | Advertising door signs on hackney Carriage and private hire vehicles | 2016 | Vehicles | 2 | Yes |  | Yes |  |

***Notes:*** *Green: Yes this harmful commodity is considered in LA policy; Amber shading: Harmful commodity included but with a more ambiguous harmful commodity definition; Red: No this harmful commodity is not considered in LA policy. Phase: (1) online searches, (2) email, (3) Freedom Of Information. ?: unknown; No#: Number(frequency) of harmful commodities considered in LA policy; Tob: Tobacco; Gamb: Gambling; Alc: Alcohol; Food: Less healthy foods; HFSS: High in Fat Salt and Sugar.*


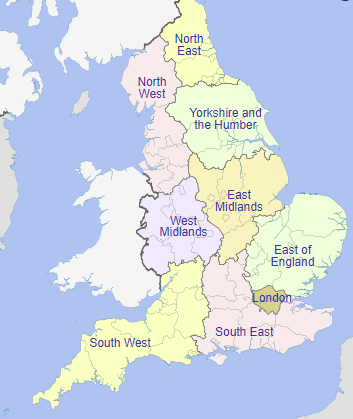
**Supplementary Material XIII.** LA policy prevalence by region

| **Region** | **Number of LAs** | **Number of policies** | **Prevalence** |
| --- | --- | --- | --- |
| South East | 70 | 22 | 31% |
| East of England | 50 | 11 | 22% |
| North West | 41 | 11 | 27% |
| East Midlands | 39 | 12 | 31% |
| London | 33 | 12 | 36% |
| West Midlands | 33 | 12 | 36% |
| South West | 33 | 15 | 46% |
| Yorkshire | 22 | 8 | 36% |
| North East | 12 | 3 | 25% |
| **Total** | **333** | **106** | **32%** |

**Supplementary Figure XIV.** LA policy prevalence by Rural/Urban classification (2011) of the Local Authority District (LAD) boundaries for 2021

| **Urban/rural Classification** | **Number of LAs** | **Number of policies** | **Prevalence** |
| --- | --- | --- | --- |
| Predominantly Urban | 179 (54%) | 64 | 36% |
| Urban with significant Rural | 61 (18%) | 16 | 26% |
| Predominantly Rural | 93 (28%) | 26 | 28% |
| **Total** | **333** | **106** | **32%** |

*LA: Local Authority; Sig: Significant; %: percentage; Prevalence: percentage of all English LAs with a policy, according to urban/rural classification.*

**Supplementary Material XV.** LA policy prevalence by LA deprivation rank (deciles and quintiles) (IMD, 2019)

| Total number of LA policies in each IMD rank decile | | |  |
| --- | --- | --- | --- |
| **Deprivation decile by rank** | **Decile** | **Number of LAs with policies** | **Prevalence** |
| Least deprived 10% | 1 | 8 | 24% |
|  | 2 | 10 | 30% |
|  | 3 | 5 | 15% |
|  | 4 | 10 | 30% |
|  | 5 | 15 | 46% |
|  | 6 | 11 | 33% |
|  | 7 | 9 | 27% |
|  | 8 | 13 | 39% |
|  | 9 | 15 | 46% |
| Most deprived 10% | 10 | 10 | 30% |
| **Total** |  | **106** |  |
|  |  |  |  |

| Total number of LA policies in each IMD rank quintile | | |  |
| --- | --- | --- | --- |
| **Deprivation quintile by rank** | **Quintile** | **Number of LAs with policies** | **Prevalence** |
| Least deprived 20% | 1 | 18 | 27% |
|  | 2 | 15 | 23% |
|  | 3 | 26 | 39% |
|  | 4 | 22 | 33% |
| Most deprived 20% | 5 | 25 | 38% |
| **Total** |  | **106** |  |
|  |  |  |  |

| Policy prevalence in the top 10 most deprived local authorities | | |
| --- | --- | --- |
| Top 10 most deprived | **According to IMD: average rank** | **Policy** |
| 1 Most Deprived LA | Blackpool | Yes |
| 2^nd^ | Manchester | Yes |
| 3 | Knowsley | No |
| 4 | Liverpool | Yes |
| 5 | Barking and Dagenham | No |
| 6 | Birmingham | No |
| 7 | Hackney | Yes |
| 8 | Sandwell | No |
| 9 | Kingston Upon Hull | No |
| 10^th^ | Nottingham | Yes |
| Total | 10 | **5 (50%)** |

**Average Rank** – this measure summarises the average level of deprivation across an area, based on the population weighted ranks of all the neighbourhoods within it.

Note: IMD 2019 does not have data for North Northamptonshire or West Northamptonshire so data for Northamptonshire is imputed for both datapoints (available in IMD 2019).

Further detail available: <https://assets.publishing.service.gov.uk/government/uploads/system/uploads/attachment_data/file/835115/IoD2019_Statistical_Release.pdf>
